# Supplementary material for: Short-Term Effects of Cooled Radiofrequency Ablation on Walking Ability in Japanese Patients with Knee Osteoarthritis
Source: J Clin Med. 2024 Nov 22;13(23):7049. doi: 10.3390/jcm13237049 (PMC11642253; doi:10.3390/jcm13237049)
Supplement: Supplementary file 1 [file jcm-13-07049-s001.zip › jcm-3329495-supplementary.pptx]

## Slide 1
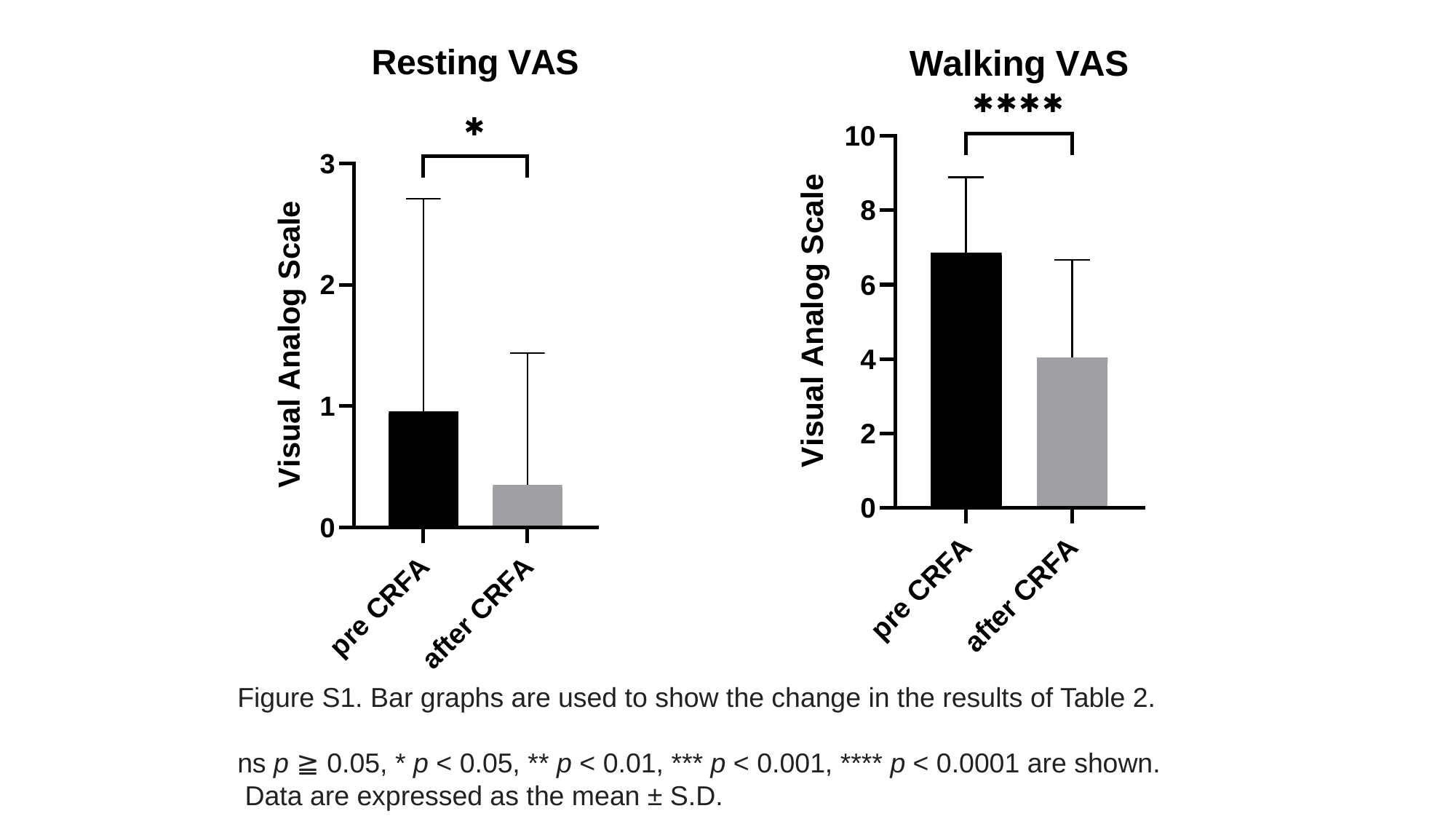

Figure S1. Bar graphs are used to show the change in the results of Table 2.
ns p ≧ 0.05, * p < 0.05, ** p < 0.01, *** p < 0.001, **** p < 0.0001 are shown.
 Data are expressed as the mean ± S.D.

## Slide 2
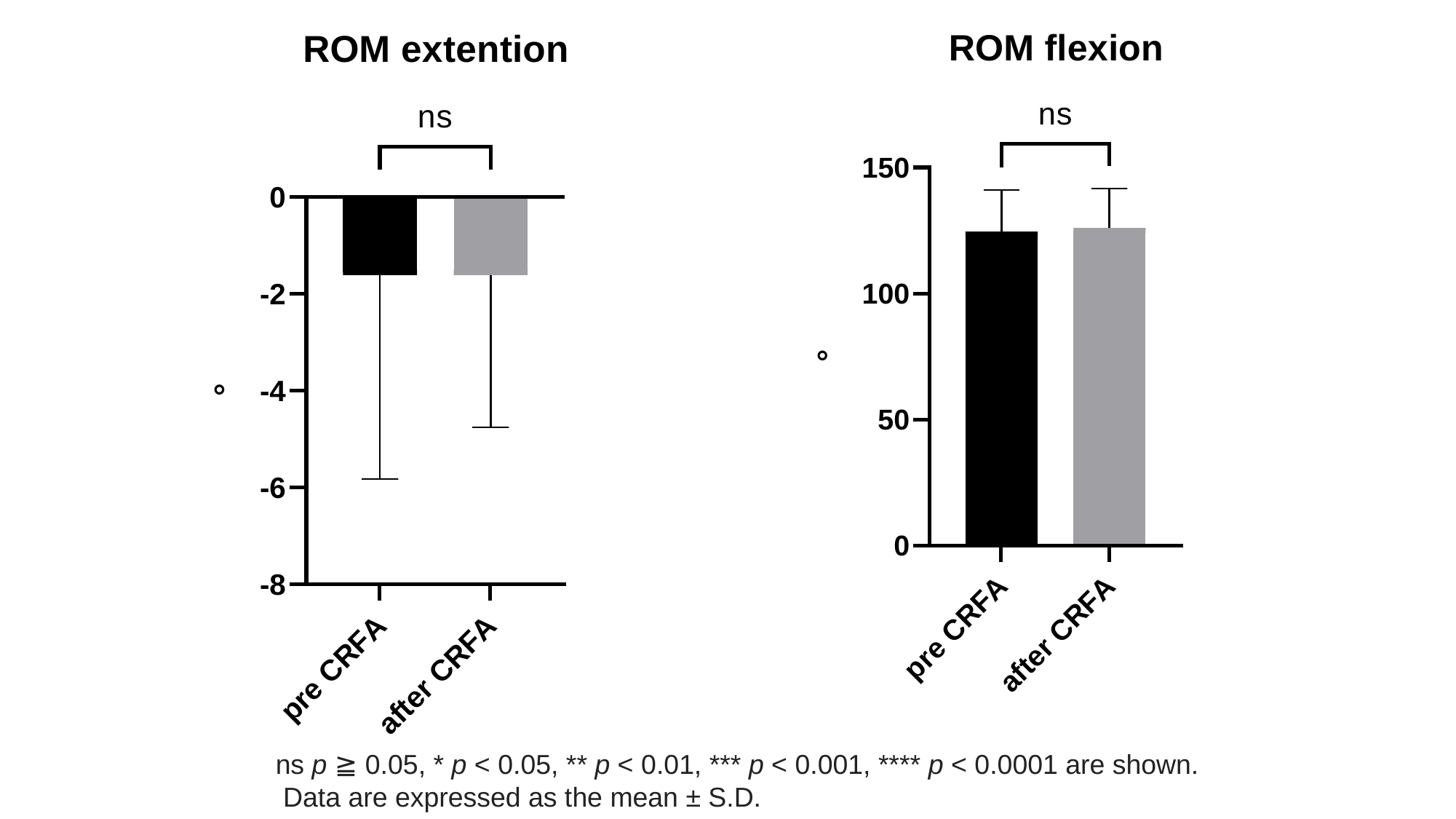

ns p ≧ 0.05, * p < 0.05, ** p < 0.01, *** p < 0.001, **** p < 0.0001 are shown.
 Data are expressed as the mean ± S.D.

## Slide 3
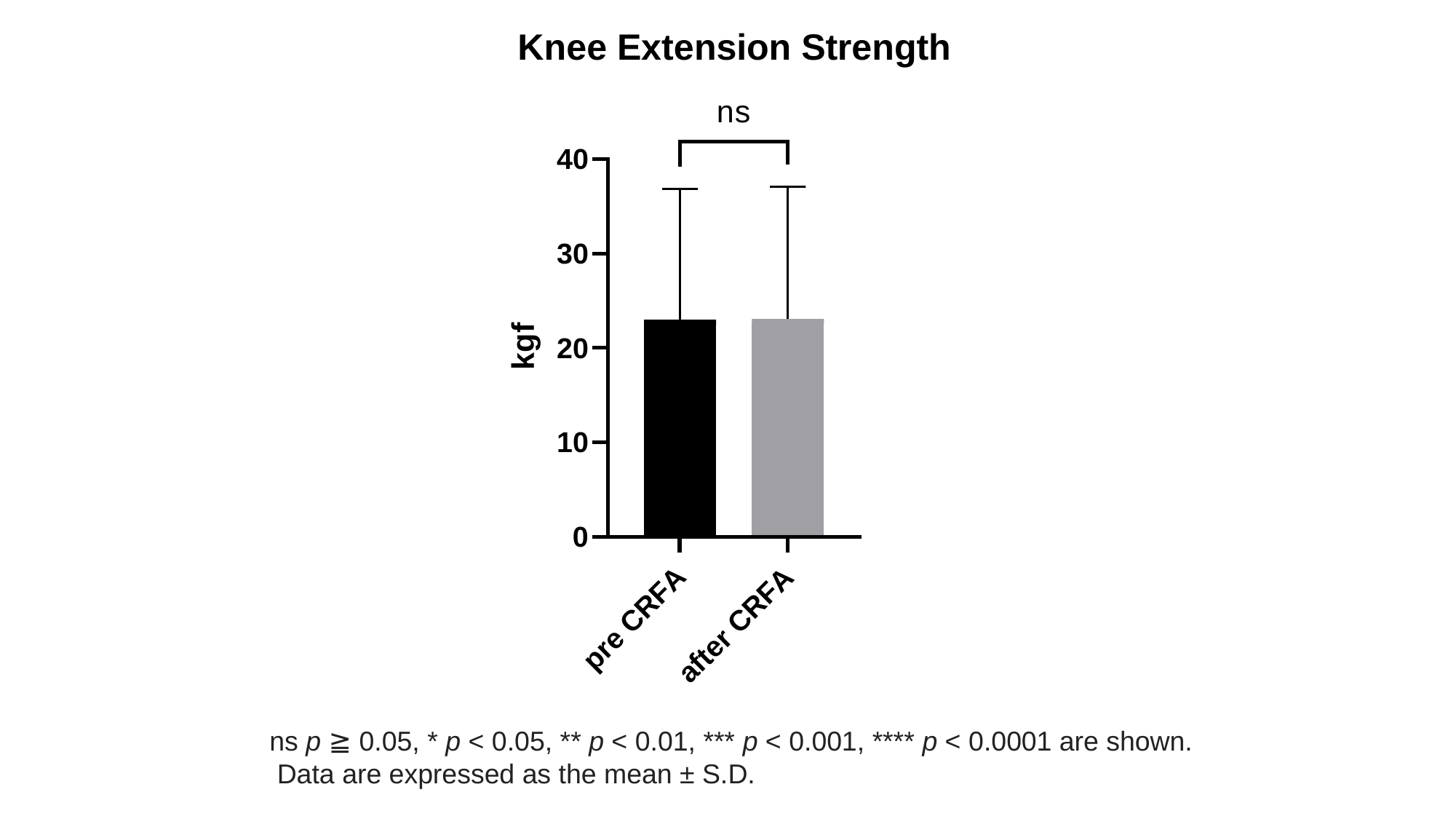

ns p ≧ 0.05, * p < 0.05, ** p < 0.01, *** p < 0.001, **** p < 0.0001 are shown.
 Data are expressed as the mean ± S.D.

## Slide 4
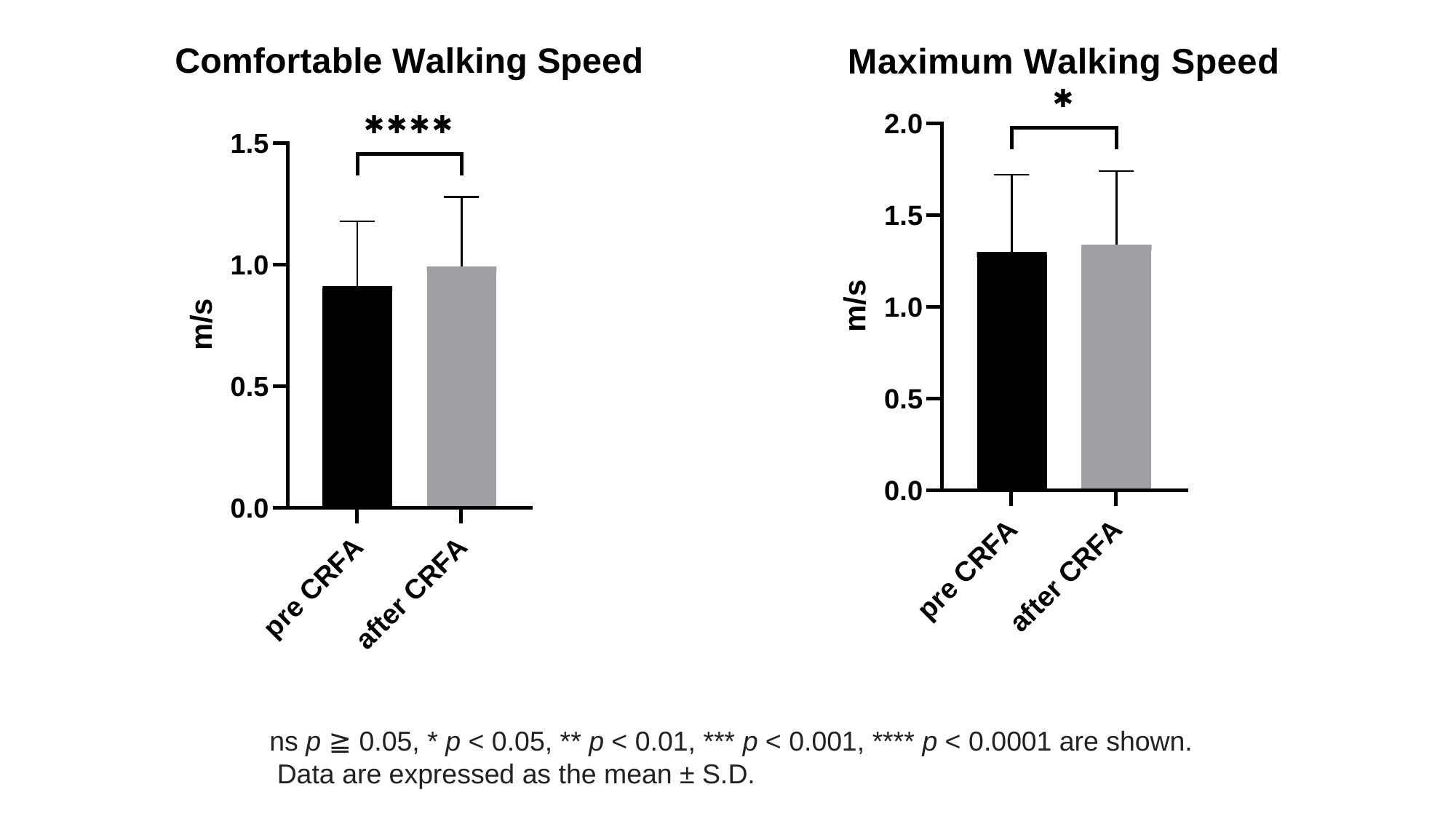

ns p ≧ 0.05, * p < 0.05, ** p < 0.01, *** p < 0.001, **** p < 0.0001 are shown.
 Data are expressed as the mean ± S.D.

## Slide 5
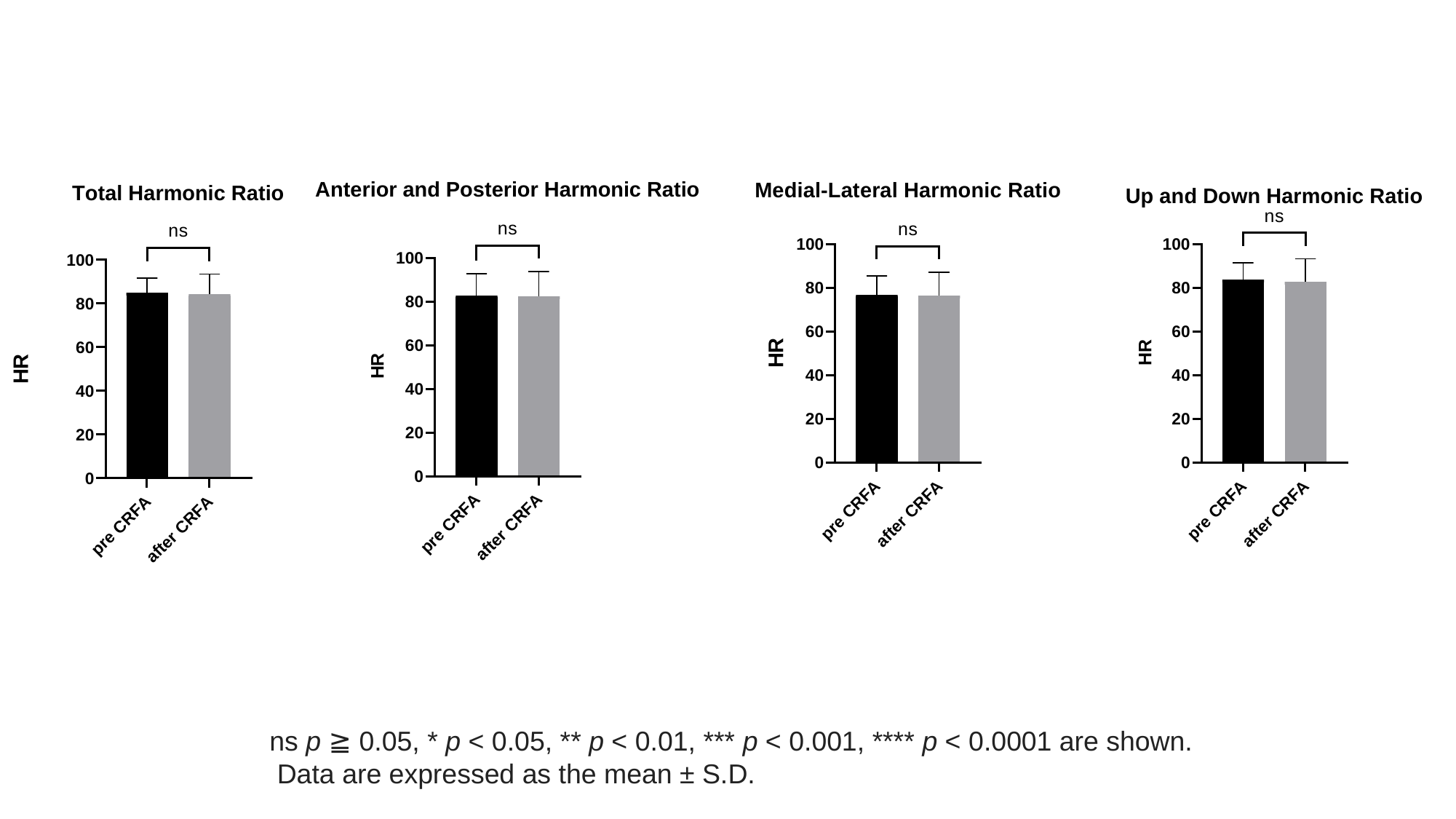

ns p ≧ 0.05, * p < 0.05, ** p < 0.01, *** p < 0.001, **** p < 0.0001 are shown.
 Data are expressed as the mean ± S.D.

## Slide 6
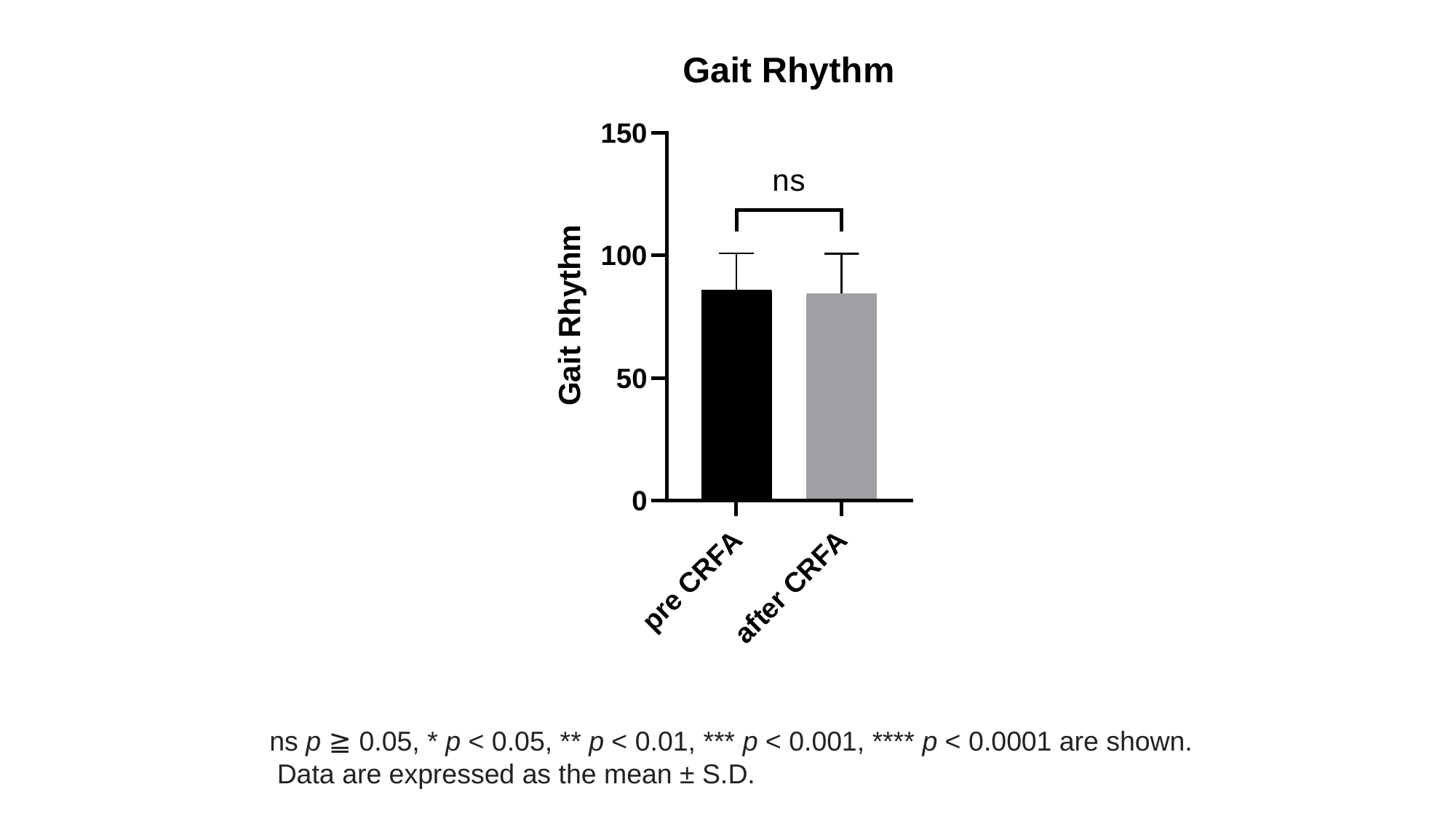

ns p ≧ 0.05, * p < 0.05, ** p < 0.01, *** p < 0.001, **** p < 0.0001 are shown.
 Data are expressed as the mean ± S.D.
